# Alpha-gliadin genes from the A, B, and D genomes of wheat contain different sets of celiac disease epitopes

**DOI:** 10.1186/1471-2164-7-1

**Published:** 2006-01-10

**Authors:** Teun WJM van Herpen, Svetlana V Goryunova, Johanna van der Schoot, Makedonka Mitreva, Elma Salentijn, Oscar Vorst, Martijn F Schenk, Peter A van Veelen, Frits Koning, Loek JM van Soest, Ben Vosman, Dirk Bosch, Rob J Hamer, Luud JWJ Gilissen, Marinus JM Smulders

**Affiliations:** 1 Allergy Consortium Wageningen, P.O. Box 16, NL-6700 AA Wageningen, The Netherlands; 2 Plant Research International, Wageningen UR, P.O. Box 16, NL-6700 AA Wageningen, The Netherlands; 3 Vavilov Institute of General Genetics, Russian Academy of Sciences, Moscow, 119991 Russia; 4 Genome Sequencing Center, Department of Genetics, Washington University School of Medicine, St. Louis, Missouri 63108, USA; 5 Leiden University Medical Center, Albinusdreef 2, E3-Q, P.O. 9600, NL-2300 RC Leiden, The Netherlands; 6 CGN, P.O. Box 16, NL-6700 AA Wageningen, The Netherlands; 7 Laboratory for Food Chemistry, Wageningen University, Bomenweg 2, NL-6700 EV Wageningen, The Netherlands

## Abstract

**Background:**

Bread wheat (*Triticum aestivum*) is an important staple food. However, wheat gluten proteins cause celiac disease (CD) in 0.5 to 1% of the general population. Among these proteins, the α-gliadins contain several peptides that are associated to the disease.

**Results:**

We obtained 230 distinct α-gliadin gene sequences from severaldiploid wheat species representing the ancestral A, B, and D genomes of the hexaploid bread wheat. The large majority of these sequences (87%) contained an internal stop codon. All α-gliadin sequences could be distinguished according to the genome of origin on the basis of sequence similarity, of the average length of the polyglutamine repeats, and of the differences in the presence of four peptides that have been identified as T cell stimulatory epitopes in CD patients through binding to HLA-DQ2/8. By sequence similarity, α-gliadins from the public database of hexaploid *T. aestivum *could be assigned directly to chromosome 6A, 6B, or 6D. *T. monococcum *(A genome) sequences, as well as those from chromosome 6A of bread wheat, almost invariably contained epitope glia-α9 and glia-α20, but never the intact epitopes glia-α and glia-α2. A number of sequences from *T. speltoides*, as well as a number of sequences fromchromosome 6B of bread wheat, did not contain any of the four T cell epitopes screened for. The sequences from *T. tauschii *(D genome), as well as those from chromosome 6D of bread wheat, were found to contain all of these T cell epitopes in variable combinations per gene. The differences in epitope composition resulted mainly from point mutations. These substitutions appeared to be genome specific.

**Conclusion:**

Our analysis shows that α-gliadin sequences from the three genomes of bread wheat form distinct groups. The four known T cell stimulatory epitopes are distributed non-randomly across the sequences, indicating that the three genomes contribute differently to epitope content. A systematic analysis of all known epitopes in gliadins and glutenins will lead to better understanding of the differences in toxicity among wheat varieties. On the basis of such insight, breeding strategies can be designed to generate less toxic varieties of wheat which may be tolerated by at least part of the CD patient population.

## Background

Wheat is an important staple food because of its characteristics of high nutritional value, technical properties and the long shelf life of the kernels. The wheat endosperm contains 8–15% protein, of which 80% is gliadins and glutenins. Hexaploid *Triticum aestivum *or bread wheat originated around 8,000 years ago from a hybridization of a tetraploid *Triticum *species with the diploid donor of the D genome *T. tauschii *[1]. The A and B genomes were most likely provided by *T. turgidum*, itself presumably formed from the wild diploid *T. monococcum *(A genome) and the donor of the B genome, a species which has so far defied conclusive identification [1]. Morphological, geographical and cytological evidence suggests *T. speltoides *(S genome) or a closely related species as the B genome ancestor. Cytogenetic research showed that the B genome is actually an altered S genome arisen by an exchange of chromosomal segments with other diploids and amphiploids, such as *Aegilops bicornis *(S^b ^genome) or *T. longissima *(S^l ^genome) [2]. According to Isidore et al. [3] polyploidization had a strong effect on intergenic sequences but the gene space was conserved.

The α-type gliadins of hexaploid *Triticum aestivum *are encoded by the *Gli-2 *locus on the short arm of the three different group 6 chromosomes [4]. Estimates for α-gliadin copy number range from 25–35 copies [5] to 100 [6] or even 150 copies [7] per haploid genome. Anderson and Greene [8] compared the sequence of 27 known cDNA and genomic clones of α-type gliadins and concluded that about half of the latter contained "in frame" stop codons and were presumably pseudogenes. The detailed constitution of the multi-gene locus is not known.

Gluten specific T cell responses in the small intestine play an important role in producing the inflammatory response in celiac disease (CD). Specific native gluten peptides can bind to HLA-DQ2/8 and induce lamina propria CD4 T cell responses causing damage of the small intestine mucosa [9,10]. Tissue damage initiates secretion of the enzyme tissue transglutaminase (tTG) for wound healing. However, this enzyme also deamidates gluten peptides, resulting in high affinity HLA-DQ2/8 binding peptides that can further increase T cell responses. Multiple T cell epitope motifs have been identified in α- and γ-gliadins as well as in glutenins [11-14], the majority of which show enhanced T cell recognition after deamidation. It also became clear that patients are generally sensitive to more than one gluten peptide. Although the DQ2/8 interaction represents the most significant association with CD so far defined, it is becoming clear that non-immunogenic gluten peptides also have an impact on the innate immunity system [15-17]. Clearly, the gluten peptide repertoire involved in CD is not yet complete.

Molberg et al. [18] and Spaenij-Dekking et al. [19] used T cell and antibody-based assays to demonstrate that a large variation exists in the amount of CD4 T cell stimulatory peptides present in α- and γ-gliadins and glutenins among diploid, tetraploid, and hexaploid wheat accessions. If this is the result of genetic differences in gluten proteins with toxic epitopes, then this would allow to design strategies for selection and breeding of wheat varieties suitable for consumption by CD patients.

In this study we first determine whether the α-gliadin genes present in the A-, B- and D-genome ancestral species are sufficiently different to attribute the ancestral genomic origin of the α-gliadin genes in hexaploid bread wheat. Secondly, we aim at understanding the diversity of CD epitopes in the α-gliadin gene family in diploid and hexaploid wheat.

## Results

### Analysis of the genomic α-gliadin genes from diploid species that represent the ancestral genomes of bread wheat

**Figure 1:**
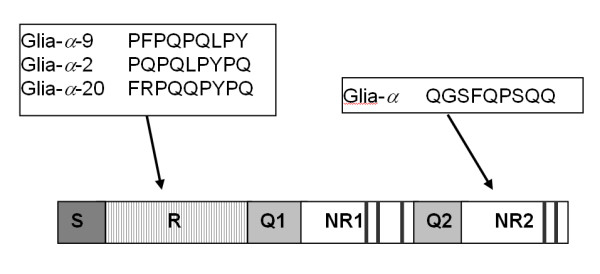
Schematic structure of an α-type gliadin protein. The protein consists of a short N-terminal signal peptide (S) followed by a repetitive domain (R) and a longer non-repetitive domain (NR1 and NR2), separated by two polyglutamine repeats (Q1 and Q2). In the non-repetitive domains five conserved cystein residues are present which are indicated with vertical lines. The T cell epitopes are shown and their approximate position is indicated.

The typical structure of the α-gliadin is depicted in Figure 1. The fact that the sequences at the 5' end (signal peptide) and 3' end of the genes are highly conserved within the α-gliadin gene family enables to obtain different members of the gene family by a PCR-based method on genomic DNA of various wheat species (Table 1). Accessions used were *Triticum monococcum*, which represents the A genome; *T. speltoides *(two accessions) and *T. longissima *that represent relatives to the B genome, and *T. tauschii *as representative of the D genome of wheat. We included these two species to represent the B genome, since these are thought to be related to the as yet unknown ancestor. This yielded 230 unique DNA clones with high similarity to known α-gliadin genes (Table 1) that were not present in the public databases. Only 31 of these sequences contained a non-interrupted full open reading frame (full ORF) α-gliadin gene. The great majority of the obtained sequences contained one or more internal stop codons or (rarely) a frameshift mutation (Table 1). We refer to the latter sequences as pseudogenes. Remarkably, no full-ORF genes but only pseudogenes from *T. longissima *were found.

**Figure 2:**
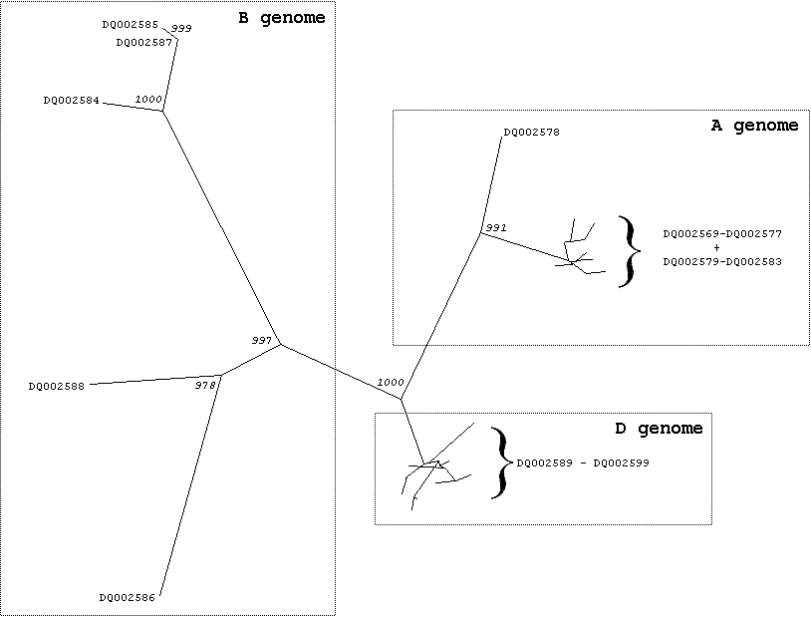
Dendrogram of a ClustalX alignment of the obtained full-ORF α-gliadin deduced proteins, which are indicated by their accession numbers (see Table 1). A PAM350 matrix and the neighbor joining method were used. Bootstrap values (of 1000 replications) are given for nodes only if they were 950 or higher.

A phylogenetic analysis of the deduced amino acid sequence of the full-ORF α-gliadin genes demonstrated a clear clustering of the sequences according to their genome of origin (Figure 2). The sequences derived from the A genome (*T. monococcum*) as well as the sequences from the D genome (*T. tauschii*) each formed a separate cluster of relatively closely related genes in the phylogenetic tree. The sequences originated from the two *T. speltoides *accessions (B genome) formed a relatively diverse cluster. All five sequences derived from the two different accessions of *T. speltoides *differed from each other. Accordingly, the fact that the B genome sequences were more diverse is not an artifact from the use of more than one representative accession.

To investigate whether the observed clustering of the sequences can be related to specific domains of the α-gliadin gene (Figure 1), the first repetitive domain (R), the first (NR1) and the second non-repetitive domain (NR2) were used separately in a phylogenetic analysis (not shown). In all cases the sequences clustered according to their genome of origin and again the A (*T. monococcum*) and D genome (*T. tauschii*) sequences clustered separately in two groups with closely related sequences whereas the sequences originating from the B genome (*T. speltoides*) formed a more diverse group with nodes of high bootstrap values. Only when using domain NR2 no significant bootstrap values were attached to the nodes within this group.

**Figure 3:**
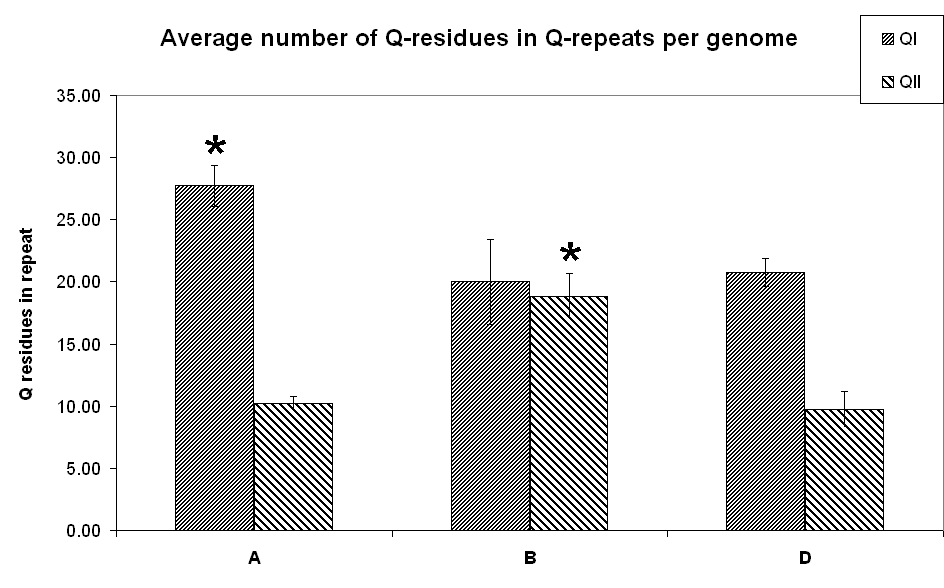
Analysis of the two glutamine repeats in the 31 obtained full-ORF α-gliadin proteins from diploid wheat species, according to the genome of origin. The average number of the glutamine residues in the first (Q1) and second repeat (Q2) are shown according to the genome of origin. The A genome (*T. monococcum*) sequences possessed a significantly higher average number of glutamine residues in the first glutamine repeat (27.7 +/- 1.7) than the B (20.0 +/- 3.4) and D (20.7 +/- 1.1) genomes did. For the second glutamine repeat, the B genome sequences demonstrated a significantly higher number of glutamine residues (18.8 +/- 1.9) than those of the other two genomes (A, 10.2 +/- 0.6; D, 9.7 +/- 1.4).

The two polyglutamine repeat domains were analyzed for differences in the average number of glutamine residues. Figure 3 shows large and also significant differences between the average lengths of the polyglutamine repeats depending on the genome of origin. The A genome (*T. monococcum*) coded for a significantly larger average number of glutamine residues in the first polyglutamine repeat than the B and D genomes. In the second polyglutamine repeat, the B genome showed a significantly larger number of glutamine residues than those of the other two genomes (Figure 3). The analysis of the repeat domains indicates that nearly all α-gliadin sequences can be assigned to one of the three genomes using only the combination of both repeat lengths.

### Analysis of the pseudogenes

**Figure 4:**
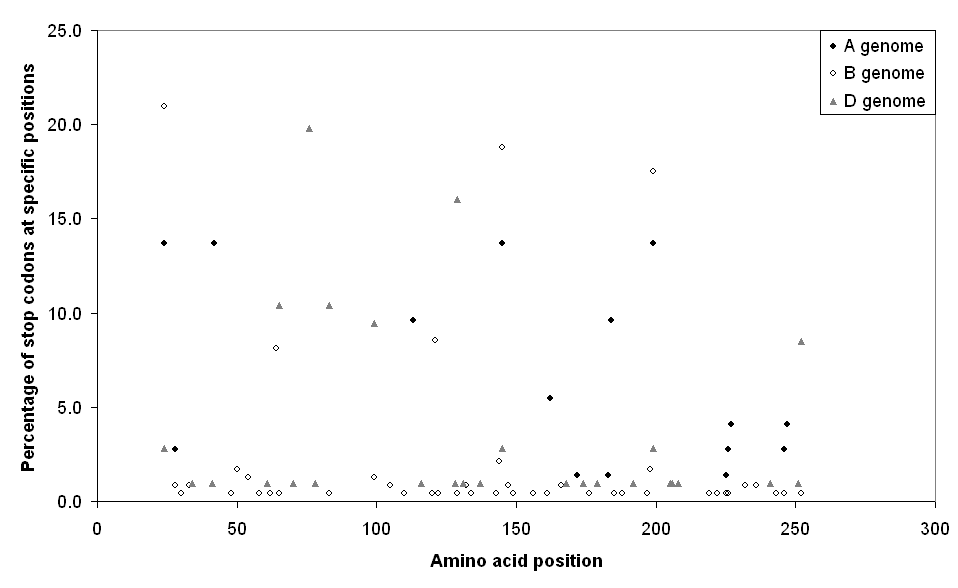
Distribution of stop codons in the pseudogenes according to the amino acid position in the sequences. The positions of the stop codons are not distributed evenly across the various diploid species. The A genome sequences have a high percentage of stop codons at positions 24, 42, 145, 199 and these four stop codons may occur jointly in one pseudogene sequence. The B genome sequences also contain a high percentage of the jointly occurring stop codons at position 24, 145 and 199 but do not contain the stop codon at position 42. The jointly occurring stop codons 24, 145 and 199 are present in a few pseudogenes originating from the D genome. Pseudogenes from the A genome may contain another pair of jointly occurring stop codons at position 113 and 184 whereas the pair at positions 64 and 121 occurs in B genome pseudogenes, and pairs of stop codons at positions 65 and 83 and at the positions 99 and 252 occur in D genome pseudogenes.

The great majority of the α-gliadin genes contained one or more internal stop codons. We refer to them as pseudogenes, although we cannot predict from the genomic data whether a subset is being expressed. A question is how and when these pseudogenes did evolve. Therefore, we determined their position in the clustering of the three genomes, and the relationship with intact ORFs in the same loci. These pseudogenes are structurally similar to the full-ORF genes. The stop codons were nearly always located at positions where the full-ORF genes contained a glutamine residue codon. A stop codon was the result of a C to T change in 77.2% of the cases when compared with the full-ORF genes, altering a CAG or CAA codon for glutamine into a TAG or TAA stop codon. In addition, we observed that 15.5% of the stop codons were caused by T to A change, altering the codon for leucine (TTG) into a stop codon (TAG). Beside these major occurring substitutions we observed some C to A, C to G, G to T, and G to A changes. Twenty of the 199 pseudogenes contained a frameshift mutation (two were obtained from *T. monococcum *(A genome), two from *T. tauschii *(D genome) and 16 from *T. longissima *and the two *T. speltoides *accessions (B genome)). The changes into stop codons were not distributed randomly across the amino acid residue positions in the sequences, and they were not distributed evenly across the various diploid species. A high percentage of stop codons occurred jointly in one pseudogene, and many pseudogenes from one species contained the same set of stop codons, suggesting that they have been duplicated after the mutations created the stop codons (Figure 4). A dendrogram of the deduced amino acid sequence of the great majority of non-frameshift pseudogenes, including the deduced amino acids downstream of the internal stop codon, closely resembled that of the full-ORF sequences. Only eleven percent of all pseudogene sequences clustered separately from the rest of the sequences of the same genome of origin.

**Figure 5:**
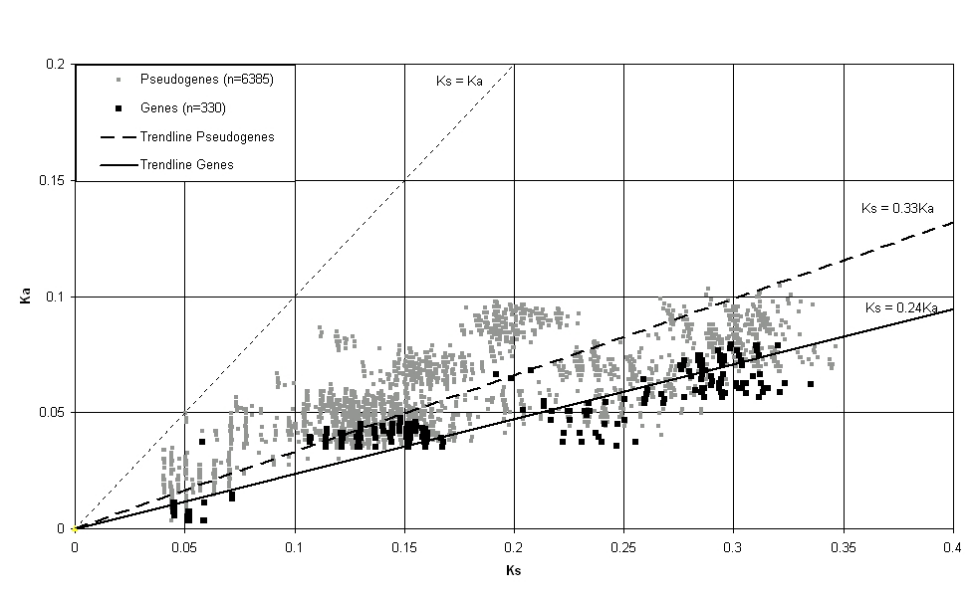
The relation of the relative numbers of synonymous substitutions (K_a_) and non-synonymous substitutions (K_s_) per site for pairwise comparisons among full-ORF α-gliadins and pseudogene sequences. The dotted line represents a K_a_/K_s _ratio of 1. Linear trendlines with the intercept set to zero are shown both for full-ORF sequences and pseudogene sequences.

To study the selection pressure on the obtained sequences the number of synonymous (K_s_) and non-synonymous (K_a_) substitutions per site were calculated from pair wise comparisons among the obtained full-ORF gene sequences and the pseudogene sequences (Figure 5). The trendlines indicated a relative excess of synonymous substitutions compared to non-synonymous substitutions and showed a stronger excess for the full-ORF genes. Consequently, the mean K_a_/K_s _ratio for the genes was significantly lower than that of the pseudogenes (*t *test; P < 0.0005), indicating the occurrence of selection.

Since the first stop codons occur in various positions in the pseudogenes, it was not feasible to select a large number of sequences of sufficient and similar length to compare the selection pressure of the sequences up to the first stop codon with that of the sequences beyond it.

### Analysis of sequences from hexaploid bread wheat

If the features described above that distinguish the α-gliadin genes from different diploid genomes, are present in hexaploid wheat in the same way, this would make it possible to assign the sequences as well as the known T cell stimulatory epitopes of α-gliadins from hexaploid wheat to one of the three loci, on chromosome 6A, 6B, or 6D. Since many hexapoid sequences are present in the public database of EMBL/Genbank/DDBJ, we tested this using the deduced amino acid sequence of these 56 full-ORF genes to build a phylogenetic tree (accession numbers are given in Table 2). The sequences of hexaploid wheat clustered into three different groups (data not shown), as did the obtained sequences from this study, separated by a very high bootstrap value (998/1000). Joint analysis together with our full-ORF sequences from diploid species showed that the three groups coincide, and this allowed us to assign each of the genes of database sequences to one of the three *Gli*-2 loci (Table 2).

### Analysis of CD-toxic epitopes

Our phylogenetic analyses show that the α-gliadin genes are distinct in their sequence conservation depending on the genomic origin. Are these patterns also being reflected in the occurrence of T cell stimulatory epitopes in the genes depending on their genomic origin? Table 3 shows the number of perfect matches in the obtained full-ORF genes and in the pseudogenes to the four epitopes studied. The results demonstrate that the set of epitopes is indeed distinct for each genome. Firstly, in the A genome (*T. monococcum*) sequences, the epitopes glia-α9 and glia-α20 were present in all 17 different full-ORF genes and in 39 (glia-α9) and in 38 (glia-α20) of the 44 pseudogenes. However, the epitopes glia-α and glia-α2 were absent. Also among the database sequences from hexaploid *T. aestivum *the sequences assigned to chromosome 6A showed the same trend in epitope occurrence (Table 2). Secondly, in the five obtained full-ORF sequences from the B genome species epitopes were completely absent except for two genes which contained the epitope glia-α only. Correspondingly, only four out of the 20 hexaploid wheat database sequences that were assigned to chromosome 6B contained epitope glia-α, whereas all others were without epitopes. Of the pseudogenes we obtained from the B genome species, 17% contained the glia-α epitope and only 3% the glia-α2 epitope, but these pseudogenes did contain the epitopes glia-α9 and glia-α20 at frequencies of 53% and 55%, respectively. Finally, in the 11 full-ORF sequences and the 64 pseudogenes obtained from the D genome, a frequent occurrence of all four different epitopes was found. This also applied to the five hexaploid wheat database sequences assigned to chromosome 6D.

**Figure 6:**
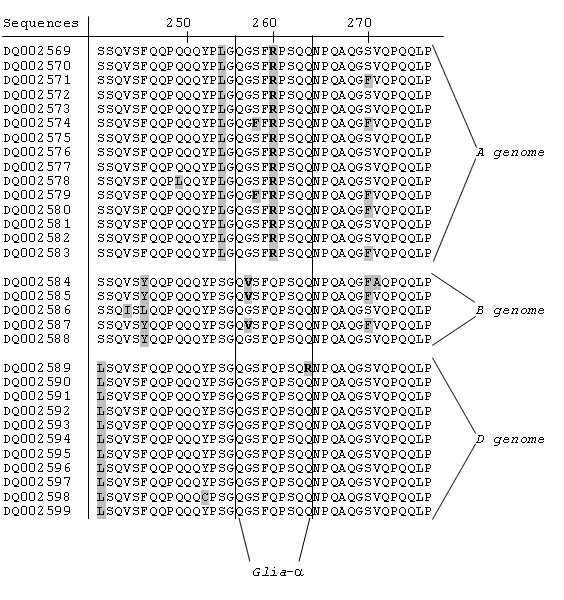
Partial detailed alignment of the obtained full-ORF α-gliadin proteins. The figure shows the disruption of epitope glia-α (QGSFQPSQQ) by a single amino acid change in all *T. monococcum *(A genome) sequences andthree of the *T. speltoides *(B genome) sequences.

**Figure 7:**
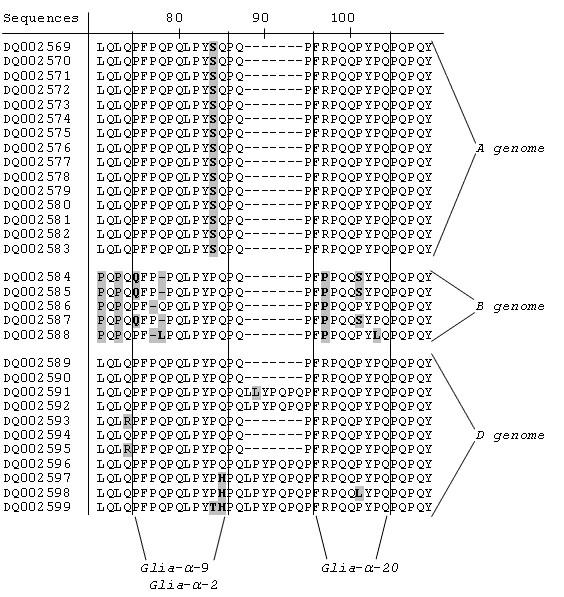
Partial detailed alignment of the obtained full-ORF α-gliadin proteins, showing the disruption of epitope glia-α2 (PQPQLPYPQ) in all *T. speltoides *(B genome), *T. monococcum *(A genome) and three *T. tauschii *(D genome) sequences. Secondly the figure shows the disruption of epitope glia-α9 (PFPQPQLPY) in the *T. speltoides *(B genome) sequences and finally the disruption of epitope glia-α20 (FRPQQPYPQ) in all *T. speltoides *(B genome) and in one *T. tauschii *(D genome) sequence.

Each epitope had its own position in the α-gliadin protein. Glia-α was in all cases present in the second non-repetitive domain (NR2), whereas glia-α2, glia-α9 and glia-α20 were all found in the first repetitive domain (R). A closer look at these sequences revealed that a single nucleotide polymorphism (SNP), which resulted in an amino acid change in a particular epitope, was present in most or all genes originating from one of the three genome. For example, Figure 6 shows that the glia-α epitope in all of the full-ORF genes derived from the A genome were disrupted at the fifth amino acid of the epitope by the presence of an arginine (R) instead of a glutamine (Q). In three B genome sequences the glia-α epitope was disrupted at the second amino acid of the epitope by the presence of valine (V) instead of a glycine (G). A detailed overview of presence of the epitopes glia-α2, glia-α9 and glia-α20 in the obtained full-ORF sequences are shown in Figure 7.

Here we show for the first time that large differences exist in the content of predicted T cell epitopes (glia-α, glia-α2, glia-α9, glia-α20) in full-ORF genes and pseudogenes from the diploid species. This phenomenon was also in hexaploid wheat. None of the diploid A genome sequences and none of the sequences from chromosome 6A in the hexaploid bread wheat contained glia-α and glia-α2 epitopes (Table 2 and 3). In contrast, the sequences from the D genome contained all four epitopes at high frequencies, both in the diploid species and in the hexaploid bread wheat. For the B genome, the five diploid and 20 hexaploid full-ORF sequences rarely contained the epitope glia-α and did not contain one of the other three epitopes. Based on this analysis, we predict that among the α-gliadin proteinss, those coded by the B genome are the least likely to stimulate CD4 T cells. Remarkably, the pseudogenes revealed the presence of all the epitopes. In these analyses we have assumed that a single amino acid substitution is sufficient to prevent such peptides from stimulating the T cells, especially since the substitutions often concern a glutamine residue. Glutamine residues can be deamidated to glutamic acid by tTG in the human gut providing the negative charges necessary to enhance binding in the DQ2 groove [9,10].

## Discussion

### Gene copy number and complexity

The diploid wheat species used in this study contain a large number of α-gliadin copies in their genome. The sequences we obtained show that the fraction of genes with in-frame stop codons is very high, ranging from 72% in the A genome species to 95% in the B genome species (Table 1). Our *in silico *comparison shows a similar situation in hexaploid wheat. The fraction of these pseudogenes appears to be higher than previously found by Anderson and Greene [8]. Analysis of the synonymous (K_s_) and non-synonymous (K_a_) substitutions in the obtained full-ORF genes and pseudogenes revealed that the pseudogenes contain more non-synonymous substitutions than the full-ORF genes. This is consistent with a reduced selection pressure on the pseudogenes. These results suggest that the majority of these sequences are not expressed (or only expressed up to the first stop codon).

### Evolution

The obtained full-ORF genes cluster together according to their genome of origin in a phylogenetic analysis. The sequence differences in the various domains of the α-gliadin genes all contribute to this clustering. The differences consisted of point mutations leading to amino acid changes at specific positions. These amino acid changes are often genome specific, suggesting that most of the duplications of this gene family have taken place after the different diploid species separated from a common ancestor. From our data, the length differences in the two glutamine repeats of the gliadin genes, which were as observed by Anderson and Greene [8], turned out to be related to the genomic origin of the genes as well. This may have occurred through the same mechanism as was found in the evolution of microsatellite repeats, where large-range mutations (duplication or deletion of a larger number of repeats through unequal crossing-over) occur infrequently, while small-step mutations (one repeat longer or shorter due to slippage) are frequent [20]. This would produce groups of similarly-sized repeats in the sequences from each genome, but the average length of each glutamine repeat could be quite different between different genomes. In addition, the large differences in the average lengths of the two repeats in the same gene indicate that unequal crossing-over between the two repeats does not take place.

Interestingly, our results clearly indicate that at least 70% of the stop codons in the pseudogenes are position and genome specific. The occurrence of stop codons at identical positions in different sequences demonstrates that pseudogene duplication has occurred. The observation that three of the stop codon positions are shared between the A and the B genome implies that some pseudogene duplications must have taken place in the common ancestor.

Based on the structural similarities to other gliadin storage proteins like the γ- and ω-gliadins [21], the α-gliadin genes on chromosome 6 are suggested to have originated from a gliadin gene on chromosome 1 through a duplication and/or translocation event [22] after the separation of wheat from rye and barley [21]. We observed that the α-gliadin genes of *T. speltoides*, and of the corresponding B genome in hexaploid bread wheat as well, are more diverse than the α-gliadin genes on the A and D genome. One explanation for this phenomenon is chromosome exchange with other species during the formation of the ancestral B genome, which is also suggested by other authors [1]. The diversity of the pseudogenes obtained from *T. speltoides *and *T. longissima *also supports this assumption. In addition, the outbreeding character of these species may have further facilitated this recombination and maintenance of diversity.

### T cell stimulatory epitopes in α-gliadin sequences

Our results indicate that, with respect to T cell toxicity as far as caused by α-gliadins, and based on currently known α-gliadin epitopes, the *Gli-2 *locus on the D genome should be considered as the most relevant.. This is in agreement with the results of Spaenij-Dekking and colleagues [19] who found the highest presence of T cell-stimulatory epitopes (glia-α-2/9) in D genome species compared to A and B genome species. In addition Molberg et al. [18] found that fragments identical or equivalent to a αG-33 mer protein fragment appear to be encoded by α-gliadin genes on the wheat chromosome 6D and are absent from gluten of diploid Einkorn wheat (A genome) and even certain cultivars of the tetraploid pasta wheat (AB genome). If these predictions are confirmed in *in vivo *studies it may follow that breeding of bread wheat for low toxicity should focus, as one of the targets, on lowering the α-gliadin proteins from the D genome. The D genome has contributed significantly to many characteristics of hexaploid wheat, including baking quality, through HMW glutenins on chromosome 1D, but there is no evidence for a specific contribution of the *Gli-2 *locus on chromosome 6D to baking quality.

Our study focused on α-gliadin genes present in the genome, and did not consider possible differences in expression among the multiple copies of α-gliadin genes. Spaenij-Dekking and colleagues [19] found large differences in T cell stimulatory epitopes in protein from different hexaploid and tetraploid accessions. Combined with our results, this may imply large differences in expression of toxic D-genome α-gliadin genes, possibly through interaction with the homologous loci on other chromosomes, as was found for ω-gliadins [23]. In that case, the mRNA pool of α-gliadins would not perfectly match the genomic composition. Alternatively, genetic differences do exist in α-gliadin sequences among hexaploid wheat cultivars.

## Conclusion

We have shown for the first time that α-gliadins from diploid *Triticum *species form distinct groups. This is reflected in large differences in the content of four T cell stimulatory epitopes (glia-α, glia-α2, glia-α9, glia-α20) in full-ORF α-gliadin genes and pseudogenes from these diploid species. Similar differences were shown to exist between the three genomes of hexaploid bread wheat. The sequence information we obtained forms a useful prerequisite for study of expressed α-gliadin mRNA and determination of both their genome of origin and their epitope content. Besides, the genetic composition of the α-gliadin loci needs to be compared across a large series of hexaploid bread wheat cultivars. As there may be more, still unknown, T cell stimulatory epitopes in all types of gluten proteins, and given that the role of the innate immune system is only beginning to be understood, it may be premature to start breeding of non-toxic wheat varieties. However, our results indicate that (re)construction of hexaploid wheat using a non-toxic D genome donor would reduce the overall T cell stimulation in CD patients.

## Methods

[GenBank: DQ002569 – DQ002798]

### DNA extraction from wheat kernels

Accessions (Table 1) were obtained from VIR, St. Petersburg, Russia (*T. longissima*) and CGN, Wageningen, the Netherlands (*T. tauschii*, *T. monococcum *and *T. speltoides*). We followed the taxonomy of *Triticum *of Morris & Sears [24]. Wheat kernels (250 mg) were grinded in liquid nitrogen and subsequently 5 ml of 65°C preheated extraction buffer (0.1 M Tris-HCl, pH 8.0; 0.5 M NaCl; 50 mM Na_2_EDTA; 1.25 % (w/v) SDS; 3.8 g/l NaHSO_4_) was added to the powder and was incubated at 65°C for 45 minutes. Then, 8 ml of chloroform/isoamylalcohol (24:1 v/v) was added. The mixture was shaken and centrifuged for 15 min at 3000 rpm. The supernatant was discarded and 8 ml ice-cold ethanol 96 % (v/v) was added. The tubes were shaken and consequently centrifuged for 10 min at 3000 rpm. The pellet was washed 2 times with 4 ml of 70 % ethanol and subsequently centrifuged for 10 min at 3000 rpm. The pellet was air-dried and dissolved in 500 μl of TE (10 mM Tris-HCl, pH 7.5 and 1 mM EDTA) + 10 μg/ml RNaseA. The solution was finally heated for 10 min at 60°C and carefully shaken.

### Amplification of α-gliadin genomic sequences

Primers to amplify α-gliadin genes from genomic DNA using PCR were designed on the conserved sequences at the 5' and 3' end of the coding region of the α-gliadin gene sequences obtained from the public database (forward primer, 1F: 5'-ATG AAG ACC TTT CTC ATC C-3', and reverse primer, 5R: 5'-GTT AGT ACC GAA GAT GCC-3'). Amplification was performed in a 25 μl reaction volume, containing 0.2 μM reverse and 0.2 μM forward primer, dNTP mix (0.25 mM each), 1 – Pfu buffer (Stratagene), 20 ng chromosomal DNA and a mixture of (1/4 v/v) Pfu DNA polymerase (Stratagene) (2.5 U/μl) and Goldstar DNA polymerase (Eurogentec) (5 U/μl). The PCR amplification utilized 3 min at 94°C followed by 25 cycles consisting of 94°C for 1 min, 55°C for 1 min and 72°C for 2 min with a final extension at 72°C for 10 min.

### Cloning and sequencing

The PCR products (lengths ranging from 900 to 1100 bp) were ligated into the pCRII-TOPO vector (Invitrogen) and subsequently used for the transformation of *E. coli*-XL1-blue cells (Stratagene). Recombinants were identified using blue-white color selection. Positive colonies were picked and grown overnight at 37°C in freeze media (36 mM K_2_HPO_4_, 13.2 mM KH_2_PO_4_, 1.7 mM trisodium citrate, 0.4 mM MgSO_4_, 6.8 mM (NH_4_)2SO_4_, 4.4 % v/v glycerol, 100 μg/ml ampiciline, 10 g/l tryptone, 5 g/l yeast extract and 5 g/l NaCl). The cloned insert was amplified directly from the culture in a PCR reaction using the M13 forward primer (5'-CGC CAG GGT TTT CCC AGT CAC GAC-3') and the M13 reverse primer (5'-AGC GGA TAA CAA TTT CAC ACA GGA-3') in 20 μl reaction volume containing 2 μl of culture. The reaction mixture consisted of the same components as well as concentrations, and utilized the same PCR program as described before. The amplified product was used in a sequencing reaction using 1F and 5R primers. Additional primers were designed on two other conserved regions of the α-gliadin gene to sequence the insert: one internal forward primer (designed on pos. 292–309), Fi1: 5'-CAA CCA TAT CCA CAA CCG-3', and one internal reverse primer (designed on position 599–615), Ri1: 5'-CA(C/T) TGT GG(A/C) TGG CTT GGC-3'. The sequence data were manually checked using the computer program Seqman from the DNAstar package. The obtained sequences were deposited in GenBank (accession numbers in Table 1).

### Phylogenetic analyses of the obtained α-gliadin clones

The deduced amino acid sequences were aligned using Clustal X (version 1.81). Phylogenetic trees were inferred by neighbour-joining (Clustal X) and parsimony (PHYLIP version 3.57c; DNAPARS) [25] and subsequently viewed using TreeView (version 1.6.6). The phylogenetic trees from the neighbour-joining (Figure 2) and parsimony analysis (not shown) were nearly identical and differed only in the organization of branches that were supported by low bootstrap values.

The deduced amino acid sequence of the full-ORF clones were analyzed without the targeting sequence (first 17 amino acids were removed) up to the former last conserved cystein residue (lengths range from 244 to 271 amino acids). In this way both primer regions were omitted. The first repetitive domain (R) (Figure 1) was analyzed from the amino acid residue on position 18 (targeting sequence was removed) until the start of the first polyglutamine repeat (length of first domain 93–105 amino acids). The first non-repetitive domain (NR1) starts with the first amino acid after the first polyglutamine repeat and ends one amino acid before the second glutamine repeat (length 68–73 amino acids). The third domain starts with the first amino acid after the second polyglutamine repeat until the former last conserved cystein residue (length 57 or 58 amino acid residues). The glutamine repeats were analyzed using the number of amino acid residues located between the beginning and the end of the polyglutamine repeat.

### Analysis on synonymous and non-synonymous substitution

The obtained nucleotide sequences were aligned codon-by-codon using Clustal W. We analysed general selection patterns at the molecular level using DnaSp 4.00 [26]. Insertions or deletions that cause a frame-shift were treated as non-synonymous substitutions. The number of synonymous (K_s_) and non-synonymous substitutions (K_a_) per site were calculated from pair wise comparisons with incorporation of the Jukes-Cantor correction, as described by Nei and Gojobori [27]. Pair wise comparisons with fewer than seven non-synonymous mutations refer to closely related sequences and contain no useful information on substitution rates. This concerned 2528 out of 9243 pair wise comparisons, which were excluded from the analyses.

### Epitope screening

All α-gliadin DNA sequences obtained in this study were translated to protein sequences and converted into FASTA format. In addition, public domain gliadin and glutenin sequences from bread wheat were extracted in FASTA-format from the Uniprot database  with the following conditions: *Triticum aestivum *and (gliadin or glutenin). The program PeptideSearch [28] was used for matching the predicted epitopes from α-gliadin with the databases described above. Only perfect matches were considered in the scoring.

## Authors' contributions

LJWJG, FK, BV, DB, RJH and MJMS initiated this study. LJWJG, BV, FK and MJMS designed the experiments, LJMS selected the plant material, MM designed the primers, SVG, JS and MM cloned and sequenced the genes, TWJMH, ES, SVG, and OV analysed the sequences, PAV and FK matched the CD epitopes, MFS performed the mutation analysis, and TWJMH, LJWJG and MJMS drafted the paper.

## Figures and Tables

**Table 1:** Number of obtained unique full open reading frame (full-ORF) and sequences with one or more stop codons (pseudogenes) from various diploid *Triticum *species. Accession numbers are given between brackets.

**Genome**	**Species, Accession**	**Full-ORF**	**Pseudogenes**	**Total**
A	*T. monococcum*, CGN 06602	15 (DQ002569 – DQ002583)	39 (DQ002600 – DQ002638)	54
B	*T. speltoides*, CGN 10682^1^	2 (DQ002584 – DQ002585)	23 (DQ002639 – DQ002661)	25
	*T. speltoides*, CGN 10684^1^	3 (DQ002586 – DQ002588)	9 (DQ002662 – DQ002670)	12
	T. longissima^1^	0	66 (DQ002671 – DQ002736)	66
D	*T. tauschii*	11 (DQ002589 – DQ002599)	62 (DQ002737 – DQ002798)	73
Total		31	199	230

^1 ^The correct annotation is S genome for *T. speltoides *and S^1 ^genome for *T. longissima *[1], but as they are here taken as closest representatives of the B genome, we will, for clarity, refer to them as B genome.

**Table 2:** Number of T cell stimulatory epitopes present in full-ORF α-gliadin genes originating from *T. aestivum *according to the deduced genome of origin. Sequences are obtainedfrom the public databases. The α-gliadin locus is on chromosome 6, but the genome (i.e., chromosome 6A, 6B, or 6D) is deduced from clustering together with sequences from the diploid species representing the ancestral genomes.

Accession number	Deduced chromosome	glia-α	glia-α2	glia-α9	glia-α20
AAA17741	6A				1
AAA34280				1	1
AAA34281					1
AAA96276				1	1
AAA96523					1
AAA96524				1	1
AAA96525				1	1
B22364					1
BAA12318				1	1
CAA10257				1	1
CAA25593				1	1
CAA26384				1	1
CAB76955				1	1
CAB76958					1
CAB76959					
CAB76960					1
CAB76961				1	1
CAB76962				1	1
CAB76963				1	1
P02863				1	1
P04721					1
S07923				1	1
T06282					1
A22364	6B				
A27319					
AAA34275					
AAA34277					
AAA34278					
AAA34279		1			
AAA34283					
AAA96522					
CAA26383					
CAA26385		1			
CAB76954					
CAB76957					
E22364					
P04723					
P04725					
P04726					
P04727		1			
S07361					
S07924		1			
T06504					
AAA34276	6D		1	1	1
AAA34282		1	1	1	
C22364					1
CAA35238		1	2	1	1
CAB76956					1
CAB76964		1	2	1	1
D22364		1	1		1
P04722			1	1	1
P04724		1	1	1	
P18573		1	2	1	1
S10015		1	2	1	1
T06498			1	1	1
T06500		1	1	1	

**Table 3:** Number of T cell stimulatory toxic epitopes present in full-ORF genes (upper panel) and pseudogenes (lower panel). N is the total number of genes used in the analyses.

**Genome**	**Full-ORF genes from**	**glia-α**	**glia-α2**	**glia-α9**	**glia-α20**	**N**
A	*T. monococcum*	0	0	15	15	15
B	*T. speltoides*	2	0	0	0	5
	*T. longissima*	-	-	-	-	-
D	*T. tauschii*	10	8	11	10	11

**Genome**	**Pseudogenes from **	**glia-α**	**glia-α2**	**glia-α9**	**glia-α20**	**N**

A	*T. monococcum*	0	0	34	34	39
B	*T. speltoides*	7	3	18	19	32
	*T. longissima*	10	0	34	35	66
D	*T. tauschii*	45	23	28	49	62
